# Identification of candidate genes that specifically regulate subcutaneous and intramuscular fat deposition using transcriptomic and proteomic profiles in Dingyuan pigs

**DOI:** 10.1038/s41598-022-06868-3

**Published:** 2022-02-18

**Authors:** Pan Zhang, Qinggang Li, Yijing Wu, Yawen Zhang, Bo Zhang, Hao Zhang

**Affiliations:** 1 National Engineering Laboratory for Animal Breeding, China Agricultural University, Beijing, 100193 China; 2 Institute of Animal Sciences and Veterinary Medicine, Anhui Academy of Agricultural Sciences, Hefei, 230031 Anhui China

**Keywords:** Genetics, Molecular biology

## Abstract

Subcutaneous fat and intramuscular fat (IMF) deposition are closely related to meat production and pork quality. Dingyuan pig, as a native pig breed in China, low selection leads to obvious genetic and phenotypic differences in the population. Individuals with extreme fat content in the population are ideal models for studying the mechanism of fat deposition. In this study, we used RNA-Seq and tandem mass tags-based (TMT) proteomics to analyze the key pathways and genes that specifically regulate subcutaneous fat and IMF deposition in Dingyuan pigs. We identified 191 differentially expressed genes (DEGs) and 61 differentially abundant proteins (DAPs) in the high and low back fat thickness (HBF, LBF) groups, 85 DEGs and 12 DAPs were obtained in the high and low intramuscular fat (HIMF, LIMF) groups. The functional analysis showed that the DEGs and DAPs in the backfat groups were mainly involved in carbohydrates, amino acids, and fatty acids metabolism, whereas the IMF groups were involved in the insulin pathway, longevity, and some disease-related pathways. We found 40 candidate genes that might tissue-specifically lipids deposition for subcutaneous and intramuscular fat. Our research provides theoretical reference materials for the improvement of fat deposition traits of local pig breeds in my country.

## Introduction

China's pig breeds are rich in genetic resources and have good meat quality. However, they also suffer from problems such as a slow growth rate, high fat deposits, and low lean meat percentages^1^. Commercial pig breeds increase the proportion of lean meat and reduce the deposition of subcutaneous fat, while the reduced intramuscular fat content at the same time results in poor flavor^2–5^. Porcine subcutaneous fat is quantified by backfat thickness, which is negatively correlated with lean meat percentage and positively correlated with intramuscular fat^6^. The IMF content plays a decisive role in the quality and flavor of pork^7^. Due to the positive genetic correlation between the two fat traits^8^, increasing the intramuscular fat content while reducing the deposition of subcutaneous fat has always been a challenge in pig breeding.

The Dingyuan pig is an indigenous pig breed distributed in Dingyuan County, Anhui Province, China. It has the advantages of high intramuscular fat, and good meat quality, but also has a low lean meat percentage and relatively slow growth rate. A large population of Dingyuan pigs was phenotyped using multi-production trait indexes, and found that the growth rate and IMF content of some individuals were similar to those of others, but their subcutaneous fat was thinner. Therefore, we believe that this population may harbor key genes that specifically regulate subcutaneous and intramuscular fat deposition, providing a good study system to investigate the molecular mechanisms regulating tissue-level differences in fat deposition.

RNA-Seq has been widely used to analyze the transcriptome profile of pig subcutaneous and intramuscular fat. Jinhua pig and Landrace pig intramuscular fat transcriptome sequencing revealed the differences in fat and protein metabolism at the transcriptional level of the two pigs^9^. Sequencing of subcutaneous fat and IMF of 100 pigs identified 215 subcutaneous fat genes and 90 IMF genes^10^. The mechanism of subcutaneous fat and IMF deposition has not only been studied in different breeds of pigs, but also in Italian Great White pigs or Duroc pigs^11^ of the same breed with different phenotypes^6^. Dingyuan pig is an excellent indigenous pig breed, but there is no relevant report yet.

In this study, we collected tissues of subcutaneous fat and the *longissimus dorsi* (LD) muscle from pigs of similar age and body weight with divergent backfat thickness and intramuscular fat content. We used RNA-Seq to study gene expression in subcutaneous and intramuscular fat simultaneously. We also used proteome analysis technology based on TMT because it has improved proteome coverage and more reliable peptide identification and quantification, which is suitable for analyzing thousands of proteins in complex biological samples^12^. Such integrated transcriptome and proteome analyses can provide insights into key functional genes and their regulatory mechanisms between different types of fat deposits in pigs.

## Results

### Summary of pig performance

The phenotypic traits of the pigs used in this study are listed in Table 1. The backfat thickness and dressing percentage of the HBF group were significantly higher than those of the LBF group (*P* = 0.001). However, the body weight, intramuscular fat content, and lean meat percentage were not significantly different between the two groups. The IMF content was significantly higher in the HIMF group than in the LIMF group (*P* = 0.006).

**Table 1:** Phenotypic data at slaughter and meat quality of Dingyuan pigs.

Groups	HBF	LBF	HIMF	LIMF
Weight (kg)	88.17 ± 5.06	90.57 ± 3.55	86.17 ± 3.29	89.67 ± 2.04
IMF (%)	6.88 ± 2.68	4.20 ± 2.05	6.30 ± 0.56**	2.97 ± 0.95
Backfat (thickness/cm)	5.53 ± 0.49**	2.53 ± 0.42	5.10 ± 1.11	3.67 ± 1.12
Dressing percentage	78.33 ± 0.01**	72.33 ± 0.01	78.10 ± 2.13	74.53 ± 3.17
Lean meat percentage	46.01 ± 5.03	50.94 ± 2.35	47.21 ± 3.33	50.42 ± 3.81

The results are expressed as mean ± SD.

*IMF* Intramuscular fat, *LBF* low backfat thickness, *HBF* high backfat thickness, *HIMF* high intramuscular fat, *LIMF* low intramuscular fat.

**indicates extremely significant (*P* < 0.01). No label indicates that the difference is not significant.

### Overview of the sequencing data

**Figure 1:**
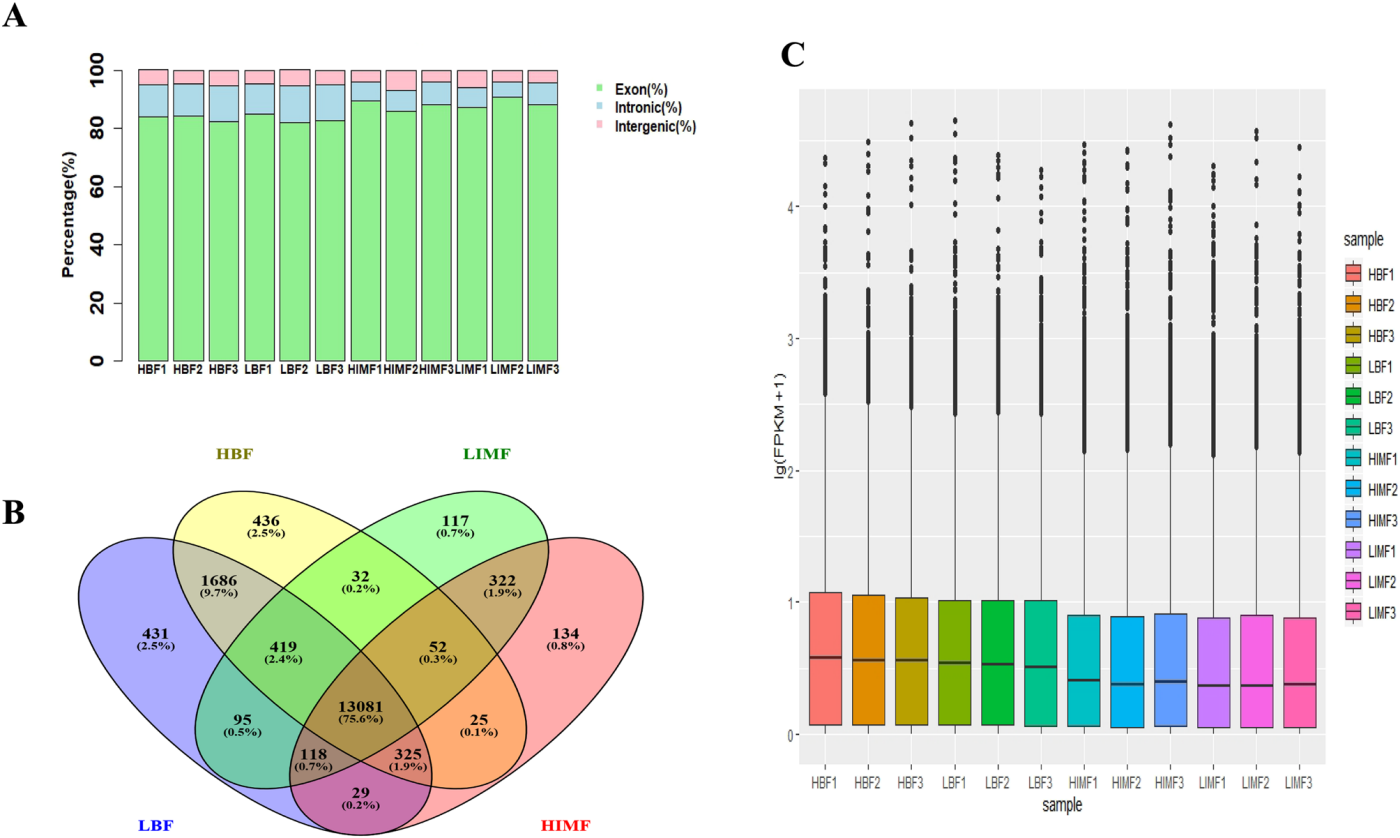
Comparison of sequencing reads and distribution of positively expressed genes. (**A**) The distribution of sequencing reads in the reference genome. The distribution of reads in the exons (green), introns (blue), and intergenic regions (pink) are shown. (**B**) Venn diagrams of expressed genes in the four groups of pigs: LBF, low backfat thickness; HBF, high backfat thickness; HIMF, high intramuscular fat; LIMF, low intramuscular fat. (**C**) Distributions of expression values of 12 samples. The boxplots show log_10_ (FPKM + 1) values of each gene from the 6 sets of RNA-Seq data. The black lines in the boxes represent the medians.

The total number of raw reads obtained by sequencing the 12 samples was 125.21 Gb and the number of clean reads was 120.87 Gb, and the average ratio of clean reads was 96.11% (94.28–97.59%; Supplementary Table S1). The transcriptome data were compared to the reference genome (Sscrofa11.1.99) and the proportion of mapped reads was > 95% (95.37–96.66%), where an average of 85.8% reads were mapped within exons, 9.2% mapped to introns, and 5.0% mapped to intergenic regions (Fig. 1A).

After quantifying the expression of protein-coding genes using fragments per kilobase million mapped reads (FPKM) analysis, we compared the expression patterns of protein-coding genes in different samples. A total of 17,302 genes (FPKM > 0.05) were obtained in the LD muscle and back subcutaneous fat tissue of Dingyuan pigs, and 13,081 genes were commonly expressed in the four groups (Fig. 1B). The gene expression distributions of protein-coding genes were similar in all samples from different tissues (Fig. 1C).

### Identification and functional analysis of differentially expressed genes

**Figure 2:**
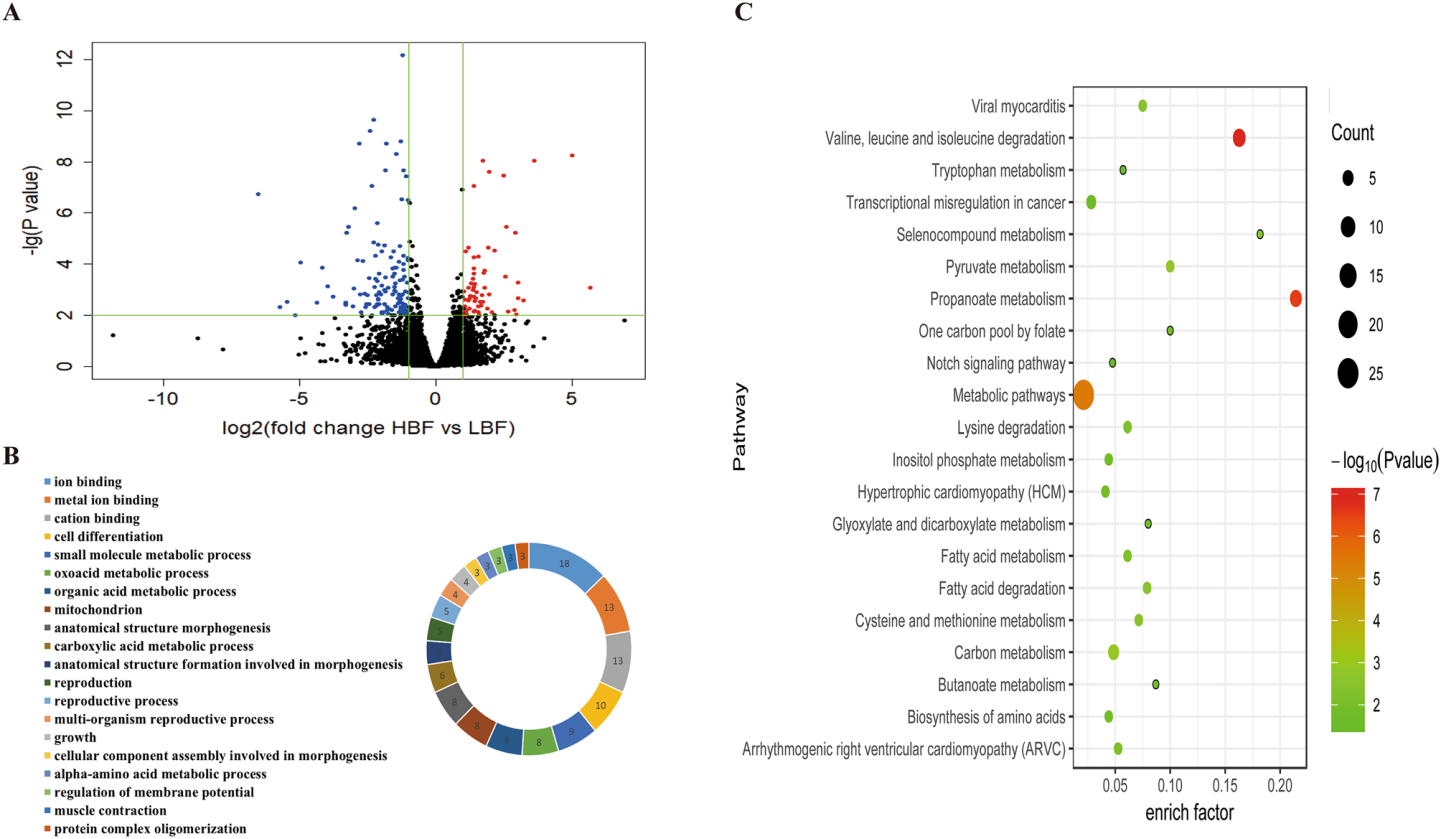
Identification and functional analysis of DEGs in the HBF and LBF groups. (**A**) Volcano plot of DEGs from samples of subcutaneous fat. The abscissa indicates log_2_FC, the ordinate indicates -lg(FDR) values, red dots indicate differential expression of upregulated genes, blue dots indicate differential expression of downregulated genes, and black dots indicate no differential expression. (**B**) GO enrichment analysis of DEGs between the HBF and LBF groups. (**C**) KEGG enrichment analysis of DEGs. The rich factor is the ratio of DEGs numbers annotated in this pathway term to the total gene numbers annotated in the same pathway term. Smaller *P*-values indicate higher significance.

A total of 191 genes were differentially expressed between the HBF and LBF groups (Supplementary Table S2), of which 63 were upregulated and 128 were downregulated in HBF (Fig. 2A). The 191 DEGs were enriched in 83 significantly enriched Gene ontology (GO) terms, which mainly involved ion binding, metal ion binding, cation binding, cell differentiation, small molecule metabolic processes, and other related biological processes (Fig. 2B, Supplementary Table S3A). Kyoto Encyclopedia of Genes and Genomes (KEGG) enrichment analysis showed that the 21 significantly enriched pathways were primarily involved in metabolism, fatty acid degradation, fatty acid metabolism, notch signaling pathway, and hypertrophic cardiomyopathy, among others (Fig. 2C). Moreover, fatty acid biosynthesis and the AMP-activated protein kinase (AMPK), PPAR, MAPK, PI3K-Akt, adipocytokine, and FoxO signaling pathways—all related to lipogenesis—were also enriched (Supplementary Table S3B).

**Figure 3:**
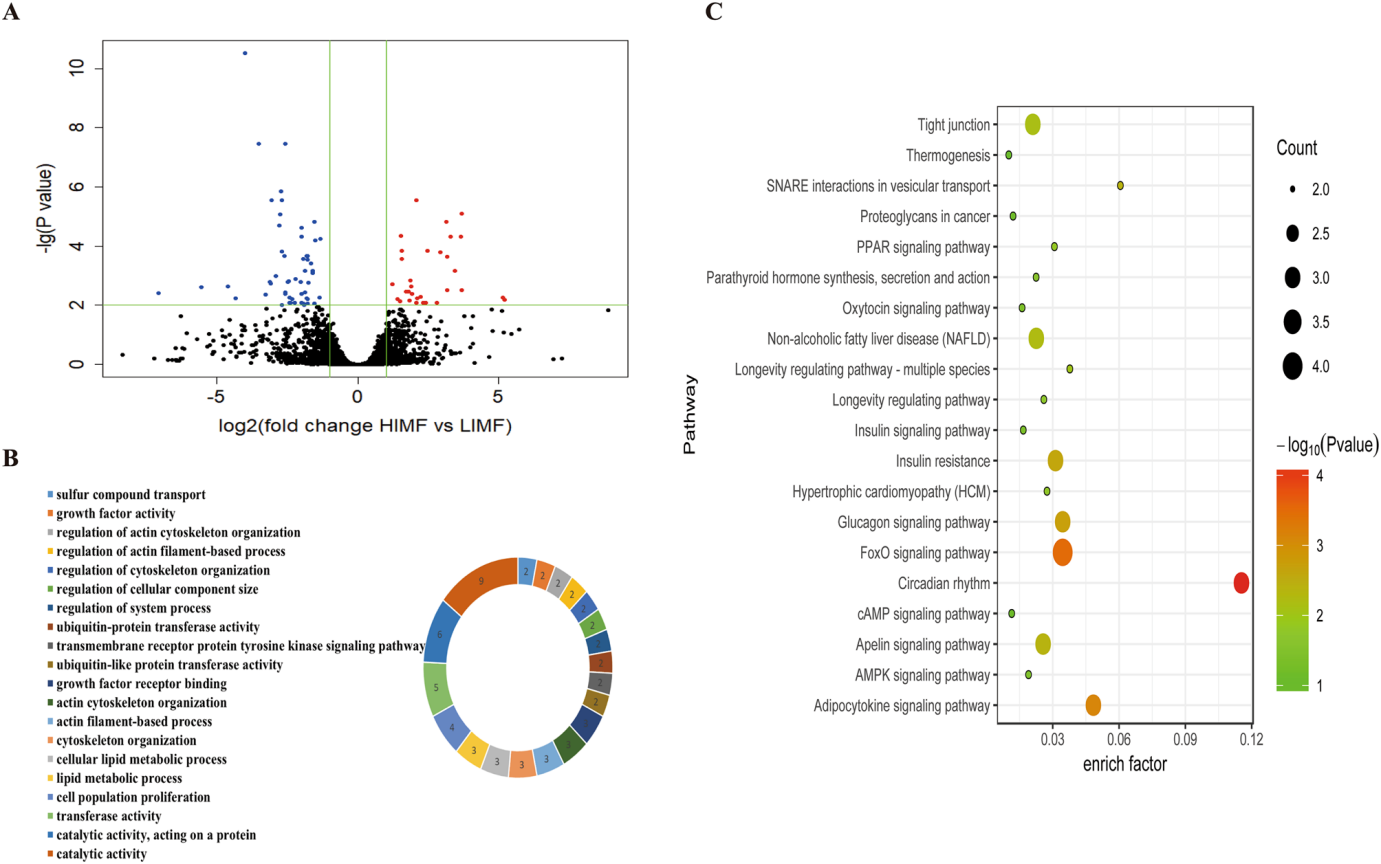
Identification and functional analysis of DEGs in the IMF group. (**A**) Volcano plot of DEGs from LD muscle samples. The abscissa indicates log_2_ fold change, the ordinate indicates -lg (FDR) values, red dots indicate differential expression of upregulated genes, blue dots indicate differential expression of downregulated genes, and black dots indicate no differential expression. (**B**) GO enrichment analysis of DEGs between the HIMF and LIMF groups. (**C**) KEGG enrichment analysis of DEGs. The enrich factor is the ratio of DEGs annotated in a pathway term to the total genes annotated in the same pathway term. Smaller *P*-values indicate higher significance.

There are 85 DEGs were identified between the HIMF and LIMF groups, including 31 upregulated and 54 downregulated genes in HIMF (Fig. 3A, Supplementary Table S4). The GO analysis identified 120 significantly enriched terms (Supplementary Table S5a), of which the top 20 most enriched genes were mainly associated with catalytic activity, cell population proliferation, growth factor receptor binding, cytoskeleton organization, and cellular lipid metabolic process, among other functions (Fig. 3B). The results of KEGG pathway analysis showed that the DEGs were involved 81 pathways and 18 were significantly enriched (Fig. 3C, Supplementary Table S5b). Among these, many were related to fat formation and metabolism, such as the FoxO, adipocytokine, PPAR, and AMPK signaling pathways.

### Comparison of DEGs from subcutaneous fat and the LD muscle

To identify the genes that specifically regulate subcutaneous and intramuscular fat deposition, we compared the DEGs from the two tissues and found 3 overlapping DEGs—keratin 80 (*KRT80*), protein kinase AMP-activated non-catalytic subunit gamma 3 (*PRKAG3*), and solute carrier family 7 member 5 (*SLC7A5*).

According to the enriched signaling pathways and expressions of DEGs, we found 21 DEGs in subcutaneous fat that may specifically regulate subcutaneous fat. Including genes related to fatty acid biosynthesis and degradation (*FASN*, *ACADSB*, and *GCDH*), amino acid metabolism (*ADHFE1*, *TKT*, and *ACAT1*), propanoate metabolism (*ECHDC1* and *ACSS3*), and carbon metabolism (*ALDH1L1* and *ALDH6A1*), as well as 11 genes (*AACS*, *SERPINE1*, *PPARD*, *UBD*, *UCP2*, *FBP1*, *CA3*, *KLF2*, *PFKFB1*, *ACP5*, *PRG4*) associated with adipocytes differentiation, butanoate metabolism, the PPAR signaling pathway, regulation of gluconeogenesis, and glucose metabolism (Table 2).

**Table 2:** Potential key candidate genes identified from the transcriptome.

Gene name	log_2_FC of HBF/LBF	FDR of HBF/LBF	log_2_FC in HIMF/LIMF	FDR in HIMF/LIMF	Gene function
*FASN*	− 2.96	1.00E−06	0.48	0.676	Metabolic pathways, fatty acid metabolism, AMPK signaling pathway, insulin signaling pathway, fatty acid biosynthesis
*CA3*	− 2.26	4.72E−03	− 0.85	0.579	Metabolic pathways, nitrogen metabolism, cellular anatomical entity, cellular process, metabolic process, binding, intracellular
*TKT*	− 1.06	5.20E−04	0.21	0.978	Metabolic pathways, carbon metabolism, amino acid biosynthesis, pentose phosphate pathway, alpha-amino acid metabolic process, sulfur compound metabolic process,
*KLF2*	1.41	5.50E−05	0.16	0.991	White fat cell differentiation
*ACAT1*	− 1.02	7.10E−05	− 0.43	0.953	Reproductive structure development, sulfur amino acid biosynthetic process, metabolic pathways, valine, leucine, and isoleucine degradation, propanoate metabolism, carbon metabolism, pyruvate metabolism
*ALDH1L1*	− 2.36	8.61E−08	− 0.91	0.913	One carbon pool by folate, One Carbon Metabolism, Folate Metabolism
*ECHDC1*	− 1.43	5.01E−09	− 0.02	0.996	Metabolic pathways, propanoate metabolism, metabolic process, catalytic activity
*ADHFE1*	− 1.85	2.19E−08	− 0.51	0.928	Reproduction, alpha-amino acid metabolic process, sulfur compound metabolic process, reproductive structure development, regulation of muscle system process, citric acid cycle, respiratory electron transport, pyruvate metabolism
*ACADSB*	− 1.19	4.80E−05	− 0.29	0.960	Metabolic pathways, valine, leucine, and isoleucine degradation, fatty acid degradation, fatty acid metabolism
*ALDH6A1*	− 1.18	4.35E−03	− 0.79	0.542	Metabolic pathways, valine, leucine, and isoleucine degradation, propanoate metabolism, carbon metabolism, inositol phosphate metabolism, beta-alanine metabolism
*AACS*	− 2.28	2.28E−10	− 0.68	0.733	Metabolic pathways, valine, leucine, and isoleucine degradation, butanoate metabolism
*SERPINE1*	1.64	3.08E−03	− 0.99	0.772	Adipogenesis, blood clotting cascade, complement and coagulation cascades
*PPARD*	1.31	5.48E−04	− 1.19	0.238	Pathways in cancer, PPAR signaling pathway, acute myeloid leukemia, Wnt signaling pathway, ion binding, metal ion binding, small molecule metabolic process
*UBD*	1.02	1.18E−03	0.07	0.995	Proteasome binding, protein ubiquitination, positive regulation of apoptotic process
*UCP2*	1.45	2.75E−03	0.28	0.974	Anatomical structure morphogenesis, reproductive structure development, cardiac muscle tissue development, cell junction organization, muscle cell development
*FBP1*	− 1.78	4.80E−03	− 3.34	–	Negative regulation of glycolytic process, regulation of gluconeogenesis, fructose 6-phosphate metabolic process, negative regulation of cell growth
*ACSS3*	− 1.20	9.09E−03	− 0.74	0.628	Metabolic pathways, propanoate metabolism
*PFKFB1*	− 3.27	6.02E−06	0.90	0.269	Regulation of glycolysis by fructose 2,6-bisphosphate metabolism, metabolism, glycolysis, glucose metabolism, focal adhesion-PI3K-Akt-mTOR-signaling pathway, carbohydrate metabolism
*GCDH*	− 1.17	2.19E−08	− 0.20	0.989	Metabolic pathways, fatty acid degradation, lysine degradation, tryptophan metabolism
*ACP5*	2.07	7.52E−03	0.92	0.953	Metabolism, metabolism of water-soluble vitamins and cofactors, NAD phosphorylation and dephosphorylation
*PRG4*	3.62	5.26E−01	− 1.39	1.948	Phospholipase-C Pathway, ERK signaling, integrin pathway, MAPK signaling
*LPL*	1.00	2.14E−03	1.16	0.102	Fatty acid β-oxidation, adipogenesis, PPAR signaling pathway, lipoprotein metabolism, triacylglyceride synthesis
*PRKAG3*	− 2.73	7.80E−03	1.88	0.001	Longevity regulating pathway, AMPK signaling pathway, apelin signaling pathway, insulin signaling pathway, oxytocin signaling pathway, non-alcoholic fatty liver disease, tight junction
*RETREG1*	− 0.74	4.03E−01	− 2.72	–	nucleolus, endoplasmic reticulum, Golgi apparatus
*PRKAG2*	− 0.50	6.82E−02	− 2.57	0.004	Vitamin digestion and absorption, thermogenesis, lipid metabolism
*SMPDL3A*	0.21	8.69E−01	− 3.53	–	Vitamin digestion and absorption, sphingomyelin phosphodiesterase activity, sphingomyelin metabolic process, cellular lipid metabolic process, membrane lipid catabolic process, sphingolipid catabolic process, phospholipid catabolic process
*IRS2*	− 0.04	9.72E−01	− 1.79	3.0E−04	Adipogenesis genes, focal adhesion-PI3K-Akt-mTOR-signaling pathway, erythropoietin activates phosphoinositide-3-kinase, IL-13 signaling pathway, signaling by type 1 insulin-like growth factor 1 receptor
*BDH1*	− 0.06	9.75E−01	− 1.93	0.009	Vitamin digestion and absorption, butanoate metabolism, metabolic pathways, ketone body catabolism, lipid metabolism
*PPARA*	− 0.58	5.02E−01	− 1.59	0.001	Vitamin digestion and absorption, cAMP signaling pathway, Hepatitis C
*GK*	0.07	9.67E−01	− 1.99	0.004	Vitamin digestion and absorption, glycerolipid metabolism, metabolic pathways
*LEP*	0.72	3.29E−01	3.17	2.3E−04	AMP-activated protein kinase Signaling, adipocytokine signaling pathway, peptide hormone metabolism, cytokine-cytokine receptor interaction

*LBF* low backfat thickness, *HBF* high backfat thickness, *HIMF* high intramuscular fat, *LIMF* low intramuscular fat, *log*_*2*_*FC* log_2_ fold change, *FDR* false discovery rate.

**Figure 4:**
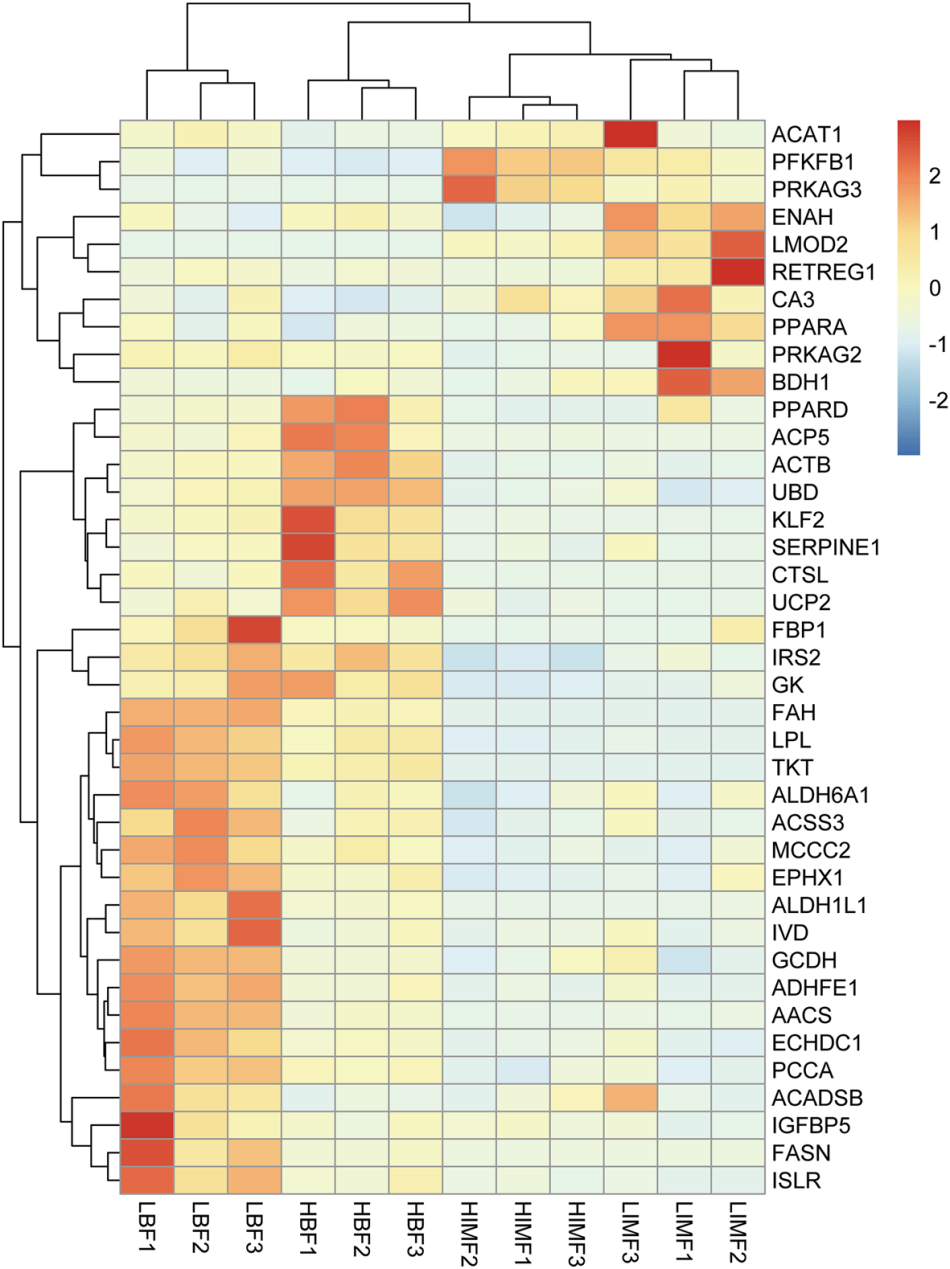
The expression levels of the candidate genes differentially expressed in the four groups of samples. Red colors represent high gene expression, blue colors represent low gene expression, and the color intensity changes with the FPKM value.

In the LD muscle, following GO and KEGG analysis, we selected 8 genes mainly involved in fat metabolism-related pathways as candidate genes for the specific regulation of intramuscular fat deposition. These included the fat synthesis gene *IRS2* (insulin receptor substrate 2), the transcription factor *PPARA* (peroxisome proliferator activated receptor alpha), the adipocyte secretory product *LEP* (leptin), genes related to fatty acid β-oxidation, the lipid metabolism-related gene *RETREG1* (reticulophagy regulator 1), *GK* (glycerol kinase), *BDH1* (3-hydroxybutyrate dehydrogenase 1), *SMPDL3A* (sphingomyelin phosphodiesterase acid like 3A), and *PRKAG2* (protein kinase AMP-activated non-catalytic subunit gamma 2). The expression levels of these candidate genes in the two tissues are shown in Fig. 4.

### Protein identification and quantification

A total of 29,519 peptides were obtained from the MS analysis of the subcutaneous fat tissues, and 4,099 proteins were identified. In contrast, 14,619 peptides and 2,178 proteins were obtained from the LD muscle. Among the identified proteins, 78.3% of those in the subcutaneous fat tissue (Supplementary Fig. S1A) and 82.3% of those in the LD muscle (Supplementary Fig. S1B) were represented by 1–10 peptides, and the molecular weight of the proteins mainly ranged from 10 to 80 kDa (Supplementary Fig. S1C,D). The coefficient of variation of > 90% of the four groups of replicates was less than 30%, indicating that our experimental samples had good biological reproducibility (Supplementary Fig. S2).

### Screening and functional classification of DAPs

**Figure 5:**
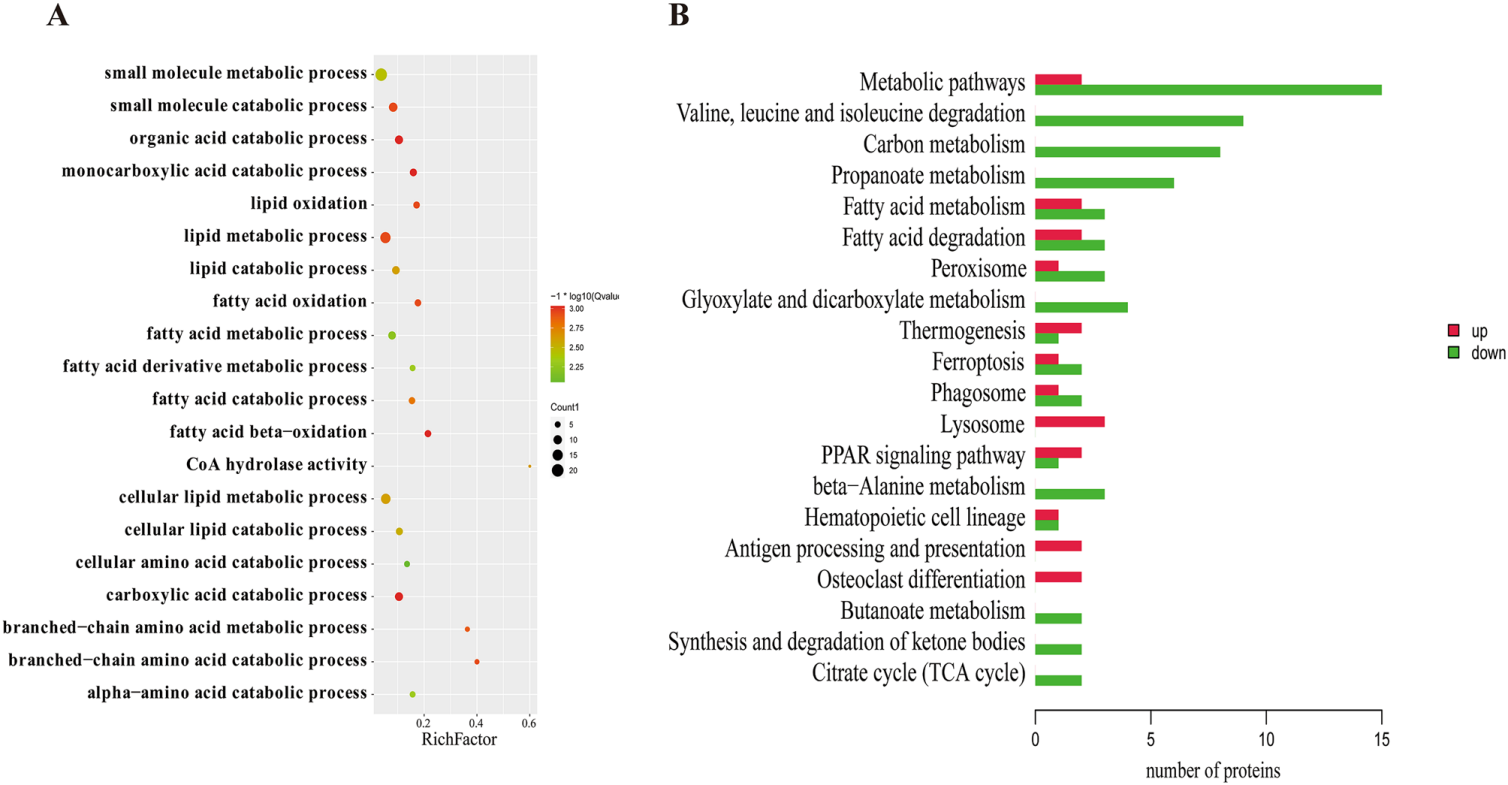
The GO and KEGG enrichment analysis of DAPs in subcutaneous fat. (**A**) GO enrichment analysis of DAPs between the HBF and LBF groups. The rich factor is the ratio of DAPs annotated in a pathway term to the total proteins annotated in the same pathway term. Smaller *P*-values indicate higher significance. (**B**) KEGG enrichment analysis of DAPs. Red colors represent upregulated protein expression and green colors represent downregulated protein expression.

In total, 62 DAPs were identified between the HBF and LBF groups, of which 22 were upregulated and 40 were downregulated (Supplementary Fig. S3, Supplementary Table S6a). The 62 DAPs were enriched in 69 GO terms. Approximately half of the 20 most significantly enriched GO terms were related to lipid and fatty acid metabolism, including the fatty acid catabolic process, fatty acid oxidation, fatty acid metabolic process, lipid oxidation, lipid metabolic process, cellular lipid metabolic process, and lipid catabolic process, among others (Fig. 5A, Supplementary Table S6b). The enriched pathways indicated by KEGG analysis mainly included fatty acid degradation, fatty acid metabolism, and the PPAR signaling pathway and metabolic pathways (Fig. 5B, Supplementary Table S6c). The 7 DAPs (ACAA2, ACAT1, ACOX1, CPT1A, ACSL4, SDHD, and IDH3A) were mainly associated with these pathways. In addition, 4 proteins—MMUT, PCCB, HMGCL, and ALDH6A1—were involved in the synthesis and degradation of ketone bodies and propanoate metabolism, and are also considered important candidate proteins (Table 3).

**Table 3:** Potential key candidate proteins identified from the proteome.

Protein name	FC of HBF/LBF	*P*-value	FC of HIMF/LIMF	*P*-value	Functional analysis
ACAA2	0.82	0.023	0.84	0.36	Propanoate metabolism, fatty acid degradation, fatty acid metabolism
ACAT1	0.71	0.043	0.96	0.87	Propanoate metabolism, fatty acid degradation, fatty acid metabolism, synthesis and degradation of ketone bodies
ALDH6A1	0.81	0.009	0.81	0.09	Valine, leucine, and isoleucine degradation, propanoate metabolism, carbon metabolism, beta-alanine metabolism, metabolic pathways
ACOX1	0.81	0.025	–	–	Propanoate metabolism, fatty acid degradation, fatty acid metabolism, PPAR signaling pathway
PCCB	0.79	0.047	0.92	0.41	Propanoate metabolism
MMUT	0.74	0.016	0.93	0.35	Propanoate metabolism
IDH3A	0.83	0.003	0.97	0.80	Carbon metabolism, metabolic pathways, citric acid cycle
HMGCL	0.79	0.048	–	–	Valine, leucine, and isoleucine degradation, synthesis and degradation of ketone bodies, peroxisome, metabolic pathways, butanoate metabolism
CPT1A	1.38	0.035	–	–	Fatty acid degradation, fatty acid metabolism, PPAR signaling pathway, adipocytokine signaling pathway, thermogenesis
SDHD	0.77	0.007	0.00	0.00	Carbon metabolism, metabolic pathways, citric acid cycle, thermogenesis, Alzheimer’s disease
ACSL4	1.20	0.006	–	–	Fatty acid degradation, fatty acid metabolism, peroxisome, metabolic pathways, PPAR signaling pathway, adipocytokine signaling pathway
TRIM55	–	–	0.81	0.022	prostaglandin metabolism
PTGR2	0.96	0.55	0.79	0.047	Metabolism of lipids, Fatty acid metabolism, Arachidonic acid metabolism,Eicosanoid metabolism via lipooxygenases
UBD	–	–	2.39	0.008	Proteasome binding, protein ubiquitination, positive regulation of apoptotic process

*LBF* low backfat thickness, *HBF* high backfat thickness, *HIMF* high intramuscular fat, *LIMF* low intramuscular fat, *log*_*2*_*FC* log_2_ fold change, *FDR* false discovery rate.

Twelve DAPs were found in the HIMF vs. LIMF groups, of which 4 were upregulated and 8 were downregulated (Supplementary Table S7a). The biological processes associated with the DAPs included developmental processes, multicellular organismal processes, protein modification processes, cellular processes, biological regulation, metabolic processes, and other biological processes (Supplementary Table S7b). In total, 14 signal pathways enriched by KEGG analysis (Supplementary Table S7c), including the PI3K-Akt signaling pathway, regulation of the actin cytoskeleton, and metabolic pathways, among others. TRIM55 (tripartite motif containing 55; involved in cellular protein metabolism), PTGR2 (prostaglandin reductase 2; involved in prostaglandin metabolism), and UBD (ubiquitin D; involved in cellular protein metabolism) were considered as candidate genes regulating IMF deposition.

### Integrated analysis of transcriptomic and proteomic data

On integrating the 191 DEGs and 62 DAPs from comparisons of the HBF and LBF groups, we found 8 overlapping genes (*ACAT1*, *ALDH6A1*, *ISLR*, *HSDL2*, *MCCC2*, *IVD*, *EPHX1*, and *PRG4*). Moreover, all 8 genes showed the same expression trends in terms of mRNA and protein. The *PRG4* gene was highly expressed in the HBF group, whereas the remaining 7 genes were low in HBF. Based on the DEGs and DAPs, a protein–protein interaction (PPI) network was established for the *Sus scrofa* database using the STRING v.10.0 online software (Supplementary Fig. S4). We found that IVD, ALDH6A1, ACSS3, ECDH1, ACADSB, ACAA2, ACSS1, HSDL2, HMGCL, PCCB, MCCC2, PCCA, AACS, ACOX1, and ACAT1 played pivotal roles in the network.

For the LD muscle tissue, among the 85 DEGs and 12 DAPs, only one overlapping gene—*LMOD2* (leiomodin 2)—was found. This gene is mainly involved in the processes of myofibril assembly and muscle structure development. The PPI network diagram shows that the DEGs and DAPs in the LD muscle group were mainly centered on the INS (Supplementary Fig. S5) and interacted with actin-related proteins and regulators such as ACTR10, ACTR1A, PFN2, and ENAH, whereas those in the other group interacted with insulin- and insulin receptor-related genes and substrate genes such as *INSR*, *IRS1*, and fat regulatory genes *LEP* and *PPARA*.

### Verification of DEGs and DAPs

**Figure 6:**
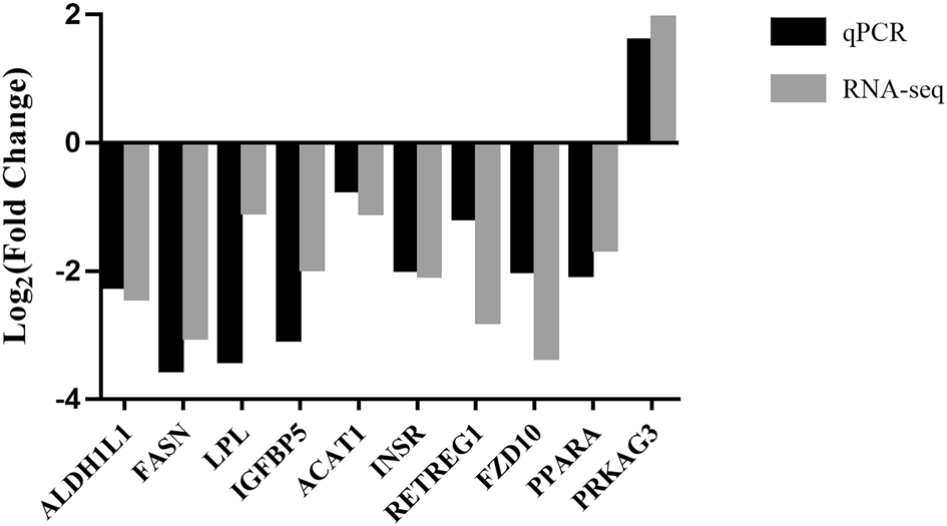
qPCR verification of genes in RNA-seq. The fold changes of the 10 genes showed that RT-qPCR results were consistent with the RNA-seq data. The fold change of qPCR was calculated as the ratio of expression between groups (HBF/LBF, HIMF/LIMF).

We selected ten DEGs (*ALDH1L1*, *FASN*, *LPL*, *IGFBP5*, *ACAT1*, *INSR*, *RETREG1*, *FZD10*, *PPARA*, *PRKAG3*) to test the validity of the RNA-Seq and TMT-based proteomic results. For this, we performed RT-qPCR using RNA samples from fat and muscle tissues of Dingyuan pigs. The expression levels of these genes in each group are shown in Supplementary Fig. S6. The 10 genes selected were differentially expressed among the groups, and the mRNA and protein expression trends of these 10 genes were concordant with those obtained by RT-qPCR. Further, fold changes of all 10 genes in the qPCR and RNA-Seq showed the same trends (Fig. 6). The results indicated that the DEGs identified by RNA-Seq and the DAPs identified by TMT-based proteomics were reliable and efficient.

## Discussion

Dingyuan pigs have high subcutaneous and intramuscular fat contents. The population is a native breed that has not undergone strong artificial selection, and contains individuals with divergent backfat thickness and intramuscular fat content. Comparing gene expression between individuals with divergent traits in the same population can reduce noise due to different genetic backgrounds. The clean reads obtained from the transcriptome mapped to > 94.2% of the genome, which is higher than the value reported in previous studies on the pig backfat and LD muscle transcriptome^9,13–15^. The current results show that our transcriptome sequencing results are of high quality. Through integrated transcriptomic and proteomic analysis, we found 8 overlapping genes in the subcutaneous fat group and only 1 overlapping gene in the LD muscle group. Due to the existence of multiple post-transcriptional regulatory mechanisms, similar results have been reported in many studies^16–19^. This prompted us to analyze integrated transcriptomic and proteomic data to better identify functional genes and their regulatory pathways.

### Comparison of enrichment function characterization

Adipose tissue is not only an important energy store but is also essential for normal carbohydrate and lipid homeostasis. In addition to storing energy, white adipose tissue can also secrete enzymes and transcription factors, and are important secretory organs in the body^20,21^. On comparing the KEGG pathways enriched by DEGs in the subcutaneous fat and the LD muscle, we found that the hypertrophic cardiomyopathy signaling pathway was the only one common to both groups. Most of the DEG-enriched pathways identified in subcutaneous fat were metabolic pathways, including the metabolic processes of various amino acids, esters, fatty acids, and other intermediate products in glucose metabolism. The DEGs in the LD muscle group participated in a wide range of signaling pathways, including insulin, ketone body synthesis and degradation, longevity, and some disease-related pathways. Our results are consistent with those of another study which reported that pathways related to adipogenesis, lipolysis, glycolysis, and fatty acid oxidation were significantly downregulated in intramuscular adipocytes compared those in adipocytes from other locations^22,23^. Previous studies have also reported the differences in the regulatory mechanisms of subcutaneous and intramuscular fat production^24^.

We separately analyzed the signaling pathways involved in the genes upregulated and downregulated in the HBF group and found that the highly expressed DEGs in the LBF group were mainly involved in fatty acid degradation and metabolism, pyruvate metabolism, pentose phosphate pathway, and other pathways related to lipid metabolism. The DEGs more highly expressed in the HBF group had functional categories related to inflammation and immunity, such as viral myocarditis, lipid and atherosclerosis, the AGE-RAGE signaling pathway in diabetic complications, and natural killer cell-mediated cytotoxicity. Human obesity is known to be associated with many diseases, including diabetes, dyslipidemia, cardiovascular disease, and cancer^25^. As an animal model for studying human obesity, pigs exhibit obesity complications similar to those of humans^26^. Therefore, it is particularly important to identify the different mechanisms of regulation of intramuscular and subcutaneous fat such that IMF content can be increased without increasing backfat deposition.

### Candidate genes affecting the production of subcutaneous adipose tissue and their regulatory changes

Among the 8 genes identified in the subcutaneous fat group, *MCCC2* and *IVD* are involved in leucine metabolism. Based on their functions, the genes *ACAT1*, *ALDH6A1*, and *PRG4* were selected as candidate proteins involved in regulating subcutaneous fat deposition in pigs. In addition to these common genes, we also identified proteins involved in fatty acid metabolism (ACAA2, ACOX1, CPT1A, and ACSL4), propionate metabolism (ALDH6A1, PCCB, MMUT), ketone body metabolism (HMGCL), and the citric acid cycle (IDH3A); the genes coding these proteins may be candidate genes for regulating subcutaneous fat in pigs. Our transcriptome proteome had very few overlapping genes, and the results indicate that there is post-transcriptional regulation of fat deposition in Dingyuan pigs. In addition, due to the limitations of sequencing technology, the number of proteins identified by the proteome was far lower than that in the transcriptome. Moreover, the number of proteins detected in the two tissues was very different, and many proteins were not detected. Therefore, we believe that DEGs that exist only in the transcriptome can also be used as important candidate genes.

In our study, *PRKAG3* was the only DEG found to participate in fat metabolism in the transcriptome of both tissues. The *PRKAG3* gene encodes the AMPK regulatory subunit γ3^27^. AMPK activity is associated with increased glucose uptake and fatty acid oxidation, as well as inhibition of glycogen synthase activity and fatty acid synthesis^28^. Two missense mutations in the pig *PRKAG3* gene—A595G (Ile199Val) and G154A (Gly52Ser)—are related to the IMF content, water-holding, and meat quality of pigs^29^, and the AMPKγ3 (R200Q) mutation results in a lack of AMP dependence and elevated basal activity of AMPK^28^. The current research on pig *PRKAG3* is focused on IMF. Based on the FPKM value of the transcriptome, we found that *PRKAG3* expression is high in muscle tissue and very low in fat tissue; therefore, the role of this gene in pig backfat deposition remains to be verified.

Our results showed that in the HBF and HIMF groups, most of the candidate genes that regulate the biological process of adipogenesis were downregulated. Lipoprotein lipase (LPL) is one of the key enzymes involved in lipid metabolism. It is the rate-limiting enzyme for the catabolism of triglycerides and can remove TG-rich, very low-density lipoproteins and chylomicrons from the blood^30,31^. Fatty acid synthase (FASN) is a key enzyme in the de novo fatty acid synthesis pathway and cell substrate energy metabolism^32^. Acetoacetyl-CoA synthetase (AACS) is an enzyme that provides acetyl units for the biosynthesis of cholesterol and fatty acids^33^. *AACS* gene expression in white adipose tissue is lower in Zucker fatty rats than in lean rats; however, in high-fat diet induced obese rats, the expression of this gene is increased^34^. ALDH1L1 and ALDH6A1 belong to the aldehyde dehydrogenases family. The *ALDH6A1* gene is involved in the metabolism of a variety of aliphatic and aromatic aldehydes produced by various endogenous and exogenous precursors, and is also an important candidate gene that affects drip loss^35^. Moreover, ALDH1L1 is an enzyme that metabolizes folate^36^. At present, there are almost no reports on the relationship between these two genes and fat deposition. However, based on the various metabolic pathways they participate in and their differential expression in the HBF and LBF groups, we speculate that they may play a role in the subcutaneous fat deposition process in pigs.

*ADHFE1* expression is highly enriched in adipose tissue and other highly metabolically active tissues, and its expression is closely related to the phenotype of mature adipocytes in vivo and in vitro^37^. The candidate genes related to subcutaneous adipogenesis that were upregulated in the HBF group included *KLF2*, *ACP5*, *SERPINE1*, *PPARD*, *UBD*, and *UCP2*. *KLF2* inhibits fat formation by negatively regulating adipocyte differentiation, but does not affect the commitment of multipotent stem cells to the preadipocytic lineage^38^. *ACP5*, which is associated with excessive fat deposition and the development of atherosclerosis in pigs^39^. *SERPINE1* (also known as PAI-1) is mainly produced in white adipose tissue and increases the cardiovascular risk in obese and diabetic patients, whereas its deficiency can prevent obesity and metabolic dysfunction^40,41^. The peroxisome proliferator-activated receptor delta gene (*PPARD*) is a key regulator of lipid metabolism, and its haplotype is associated with pig backfat thickness^42^. Studies in C2C12 myoblasts have shown that overexpression of *PPARD* inhibits myotube formation and enhances adipocyte differentiation^43^. In mice, knockout of the *UBD* gene prevents the development of age-related obesity while prolonging lifespan and vitality^44^. *UCP2* participates in the oxidation of mitochondrial substrates and plays an important role in metabolic pathways^45,46^. Studies have reported that some SNPs in *UCP2* are related to obesity and type 2 diabetes^47–49^. Among these differential genes, genes that are positively related to adipogenesis were down-regulated (e.g., *FASN*), and some were upregulated (e.g., *ACP5*), indicating that fat metabolism in the body is a dynamic and balanced process.

### Candidate genes affecting the production of IMF and their regulatory changes

In the transcriptome of the IMF group, we found many enriched pathways related to lipid metabolism. Based on these pathways, we identified genes that may regulate intramuscular fat deposition. *RETREG1*, also known as *pFAM134B*, promotes the accumulation of subcutaneous fat in pigs by increasing the mRNA levels of *PPARγ*, *FAS*, and *ACC*, and reducing the levels of *ATGL* and *HSL*^50^. *PRKAG2* is an important regulator of cardiac metabolism^51^; an SNP of *PRKAG2* is associated with insulin sensitivity and is essential for obesity, triglycerides, and HDL cholesterol^52^. SMPDL3A—a recently identified phosphodiesterase—plays a role in cholesterol and fat metabolism by regulating the liver X-receptor^53^. The insulin receptor substrate 2 (IRS2) is the first downstream effector of the insulin receptor. Studies have found that the absence of IRS2 in myeloid cells improves glucose homeostasis and enhances resistance to metabolic dysfunction induced by a high-fat diet^54^. In addition, the proteins LEP^55^, GK^56^, and PPARA^57^—reported to be associated with obesity or adipose-related biological processes—were also included in our results.

The purpose of our experiment was to identify genes that specifically regulate subcutaneous and intramuscular fat in pig with extreme backfat and IMF. Interestingly, some of the DEGs and DAPs identified in the backfat groups in this study. For instance, *UBD* expression was upregulated in our HBF transcriptome and in the proteome of the HIMF group. Studies have shown that *UBD* has a positive regulatory effect on intramuscular and subcutaneous fat, and that inhibition of *UBD* in intramuscular and subcutaneous adipocytes results in the inhibition of cell proliferation and lipid droplet production^58^.

From our results, it is worth mentioning that the *RETREG1* gene was downregulated in the HIMF group. Although it has been suggested that this gene has an inhibitory effect on intramuscular fat deposition in Dingyuan pigs, studies have shown that it also promotes subcutaneous fat deposition. The opposing roles of genes in the two types of adipose tissue are beneficial for the selection of high-quality pigs, and their functions can be explored through further studies.

## Materials and methods

### Animal ethics compliance

All animal work was conducted following the guidelines for the care and use of experimental animals. The Animal Welfare Committee of the State Key Laboratory for Agro-Biotechnology of the China Agricultural University approved all procedures for animal care (approval number, SKLAB‐2012‐04‐07). The study was carried out in compliance with the ARRIVE guidelines.

### Animals and samples

The Dingyuan pigs were raised in the Ankang Agriculture and Animal Husbandry Company, Dingyuan County, Anhui Province, with consistent feeding conditions. Over 200 unmated sows that were 10 months old and showed normal growth were selected and their body weight and live backfat thickness were measured using a platform scale and a B-ultrasound instrument (SSD-500V, ALOKA, Japan). Based on the measurements, 12 individuals with similar body weights were selected such that six had extremely high live backfat thickness and six had extremely low live backfat thickness. The pigs were slaughtered to measure the carcass weight, backfat thickness and lean meat percentage. The LD muscle and back subcutaneous fat tissues were collected, immediately frozen in liquid nitrogen, and stored at − 80 °C.

### Measurement of intramuscular fat

A fresh sample of the LD muscle tissue was cut into pieces, the connective tissue and fascia were removed, and the remaining tissue was homogenized. The LD muscle samples were then dried to a constant weight in an oven and the dried samples were ground into powder. The Soxhlet extraction method was used to determine IMF content in LD muscle tissue.

Based on backfat thickness and IMF content, subcutaneous fat samples were divided into high backfat thickness (HBF, n = 3) and low backfat thickness (LBF, n = 3), and LD muscle samples were divided into the high intramuscular fat (HIMF, n = 3) and low intramuscular fat (LIMF, n = 3) groups.

### Library preparation and sequencing

Total RNA was extracted from the LD muscle and back subcutaneous fat tissues using the TRIzol (Invitrogen, Carlsbad, CA, USA) method. Isolated total RNA was quantified (Nanodrop, ND2000) and quality controlled with typical curves (Agilent, Bioanalyzer 2100). The RNA integrity number (RIN) values of all samples were above 7, and 2.5 µg of total RNA was used to construct the cDNA library (TruSeq RNA Sample Preparation Guide, Illumina Inc., San Diego, CA). The Illumina NovaSeq6000 platform **(**Illumina, San Diego, CA, USA) was used for transcriptome sequencing, and the sequencing read length was paired-end 150 bp. The obtained raw data were filtered to clean data with fastp (version 0.12.3) by removing reads containing adapters, low-quality reads (Q-value < 19), and reads with an N content > 5%. Clean reads were mapped to the reference pig (*Sus scrofa*) genome version scrofa.Sscrofa11.1.99^59^ (http://ftp.ensembl.org/pub/release-99/fasta/sus_scrofa/dna/Sus_scrofa.Sscrofa11.1.dna.toplevel.fa.gz) using the HISAT2 (V2.0.5) software for alignment. We used the Cufflinks (V2.2.1) software to calculate FPKM values to represent gene expression levels. The DESeq2 (V1.25.9) was used to analyze the differences between groups, and the *P*-values were adjusted using the Benjamini–Hochberg method. Differentially expressed genes (DEGs) were defined as those with false discovery rate (FDR) < 0.01 and |log_2_ fold change|≥ 1.

### Protein sample preparation and TMT tag labeling

The samples used for the proteomic analyses were the same as those used for RNA-Seq. An appropriate amount of sample tissue was added to the protein lysis solution (Mammal Tissue Total Protein Extraction Kit AP0601-50, Invent Biotechnologies, Inc., Plymouth, MN, USA). After sonication, the samples were placed on ice for 20 min, centrifuged at 10,000× g at 4 °C for 30 min, transferred to a new tube, and stored at − 80 °C. Protein concentrations of the samples were determined using a BCA kit (Thermo Fisher Scientific), and protein quality was detected by SDS-PAGE. The peptides were labeled with TMT isobaric tags (Thermo Fisher Scientific) at room temperature for 1 h.

The TMT‐labeled samples were fractionated using a high-pH peptide fractionation protocol. Each sample was fractionated into 60 fractions using a step gradient of 0–95% acetonitrile. Next, the 60 fractions were pooled into 10 fractions in a non-contiguous manner. Each sample was separated using a high-performance liquid chromatography (HPLC) system. The chromatographic column was balanced with 95% solution A (98% H_2_O, pH10), loaded onto the mass spectrometer pre-column using the autosampler, and then separated by an analytical column. The separated samples were analyzed using a Q Exactive Plus mass spectrometer (Thermo Fisher Scientific).

After mass spectrometry analysis, the Proteome Discoverer 2.4 software (Thermo Fisher Scientific) was used to process all the original files against the Sus_scrofa.Sscrofa11.1.pep.all.fa database. The peptide confidence was set to a high level (*q*-value < 0.01) for peptide filtering. Quantification of experimental bias was set to normalize the total peptide amount. FC ≥ 1.2, FC ≤ 0.833, and *P*-value ≤ 0.05 were set as thresholds for identifying differentially abundant proteins (DAPs).

### Functional annotation of DEGs and DAPs

GO and KEGG pathway enrichment analysis of the DEGs was performed using the KOBAS 3.0 enrichment analysis tool (https://kobas.cbi.pku.edu.cn/anno_iden.php). Functional analysis of DAPs was conducted using the UniProt ftp database and the KEGG PATHWAY database^60^. The Pathway Maps tool was used to enrich the pathways, and *P*-values were calculated using R software based on a hypergeometric distribution, with the default database being used as the background.

### Validation of important candidate genes

To confirm the DEGs identified in RNA-Seq and the DAPs identified in TMT-based proteomics, qRT-PCR was performed on 10 genes with the same samples as those used for sequencing to measure their expression levels in different groups. The primer sequences used are listed in Supplementary Table S8. Quantitative real-time PCR (qPCR) reactions were performed using the SYBR Green qPCR SuperMix (Transgen, Beijing, China) using a Bio-Rad CFX96 System (Bio-Rad, Hercules, CA, USA) with a reaction volume of 20 μL. A cDNA pool of all samples was used for calibration and the experiment was performed in triplicate for each sample. Gene expression levels were calculated using the 2^−ΔΔCt^ method.

### Statistical analysis

The differences in slaughter traits and gene expression levels were analyzed using the t-test in SPSS (version 21.0; IBM Corp. Released 2012. IBM SPSS Statistics for Windows, Armonk, NY, USA). Graphs were prepared using GraphPad Prism (version 7), and data are presented as the mean ± standard error.

## Supplementary Information

Supplementary Figures.

Supplementary Table S1.

Supplementary Table S2.

Supplementary Table S3.

Supplementary Table S4.

Supplementary Table S5.

Supplementary Table S6.

Supplementary Table S7.

Supplementary Table S8.

## Data Availability

All RNA-Seq data were deposited in the Gene Expression Omnibus under accession number GSE179457 (https://www.ncbi.nlm.nih.gov/geo/query/acc.cgi?acc=GSE179457). The mass spectrometry proteomics data have been deposited to the ProteomeXchange Consortium via the PRIDE partner repository with the dataset identifier PXD027061.The datasets supporting the conclusions of this article are included within the article and its additional files.

## Abbreviations

DAP: Differentially abundant protein
DEG: Differentially expressed gene
FDR: False discovery rate
FPKM: Fragments per kilobase million
GO: Gene ontology
HBF: High backfat thickness group
HIMF: High intramuscular fat group
IMF: Intramuscular fat
KEGG: Kyoto encyclopedia of genes and genomes
LBF: Low backfat thickness group
LD: *Longissimus dorsi*
LIMF: Low intramuscular fat group
log_2_FC: Log_2_ fold change
qPCR: Quantitative real-time PCR
TMT: Tandem mass tags
