# Supplementary material for: Identification of candidate genes that specifically regulate subcutaneous and intramuscular fat deposition using transcriptomic and proteomic profiles in Dingyuan pigs
Source: Sci Rep. 2022 Feb 18;12:2844. doi: 10.1038/s41598-022-06868-3 (PMC8857214; doi:10.1038/s41598-022-06868-3)
Supplement: Supplementary file 1 — Supplementary Figures. [file 41598_2022_6868_MOESM1_ESM.pdf]

Identification of candidate genes that specifically regulate subcutaneous and intramuscular fat deposition from transcriptomic and proteomic profiles in Dingyuan pigs

Figure S1

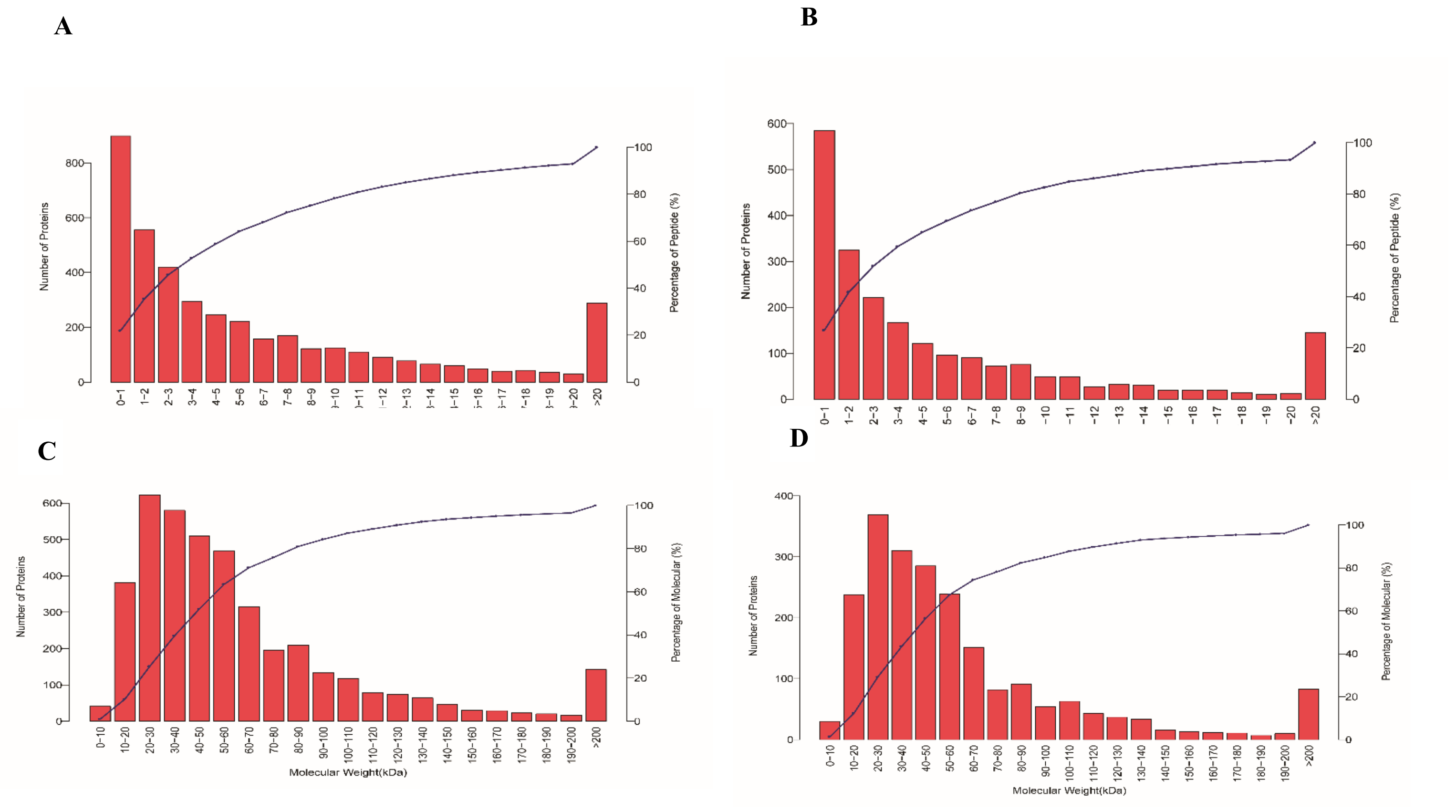

Figure S1. Basic information on proteins identification. (A) Distribution of peptide numbers identified in HBF and LBF groups. (B) Distribution of peptide numbers identified in HIMF and LIMF groups. (C) The relative molecular mass distribution map of the identified proteins in the HBF and LBF groups. (D) The relative molecular mass distribution map of the identified proteins in the HIMF and LIMF groups.

Figure S2

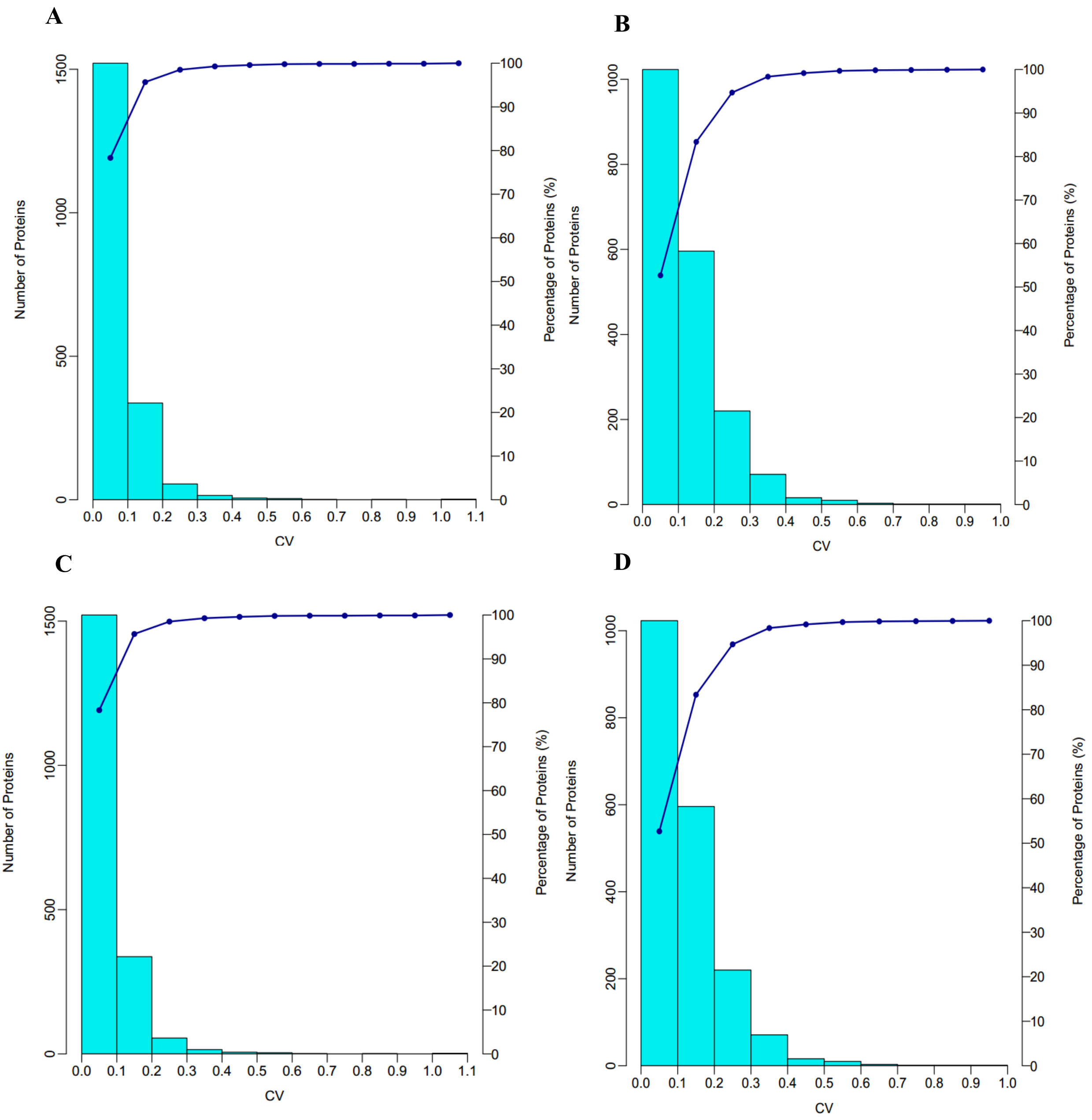

Figure S2. The coefficient of variation (CV) of proteins in the replicates of the four groups. (A) Coefficient of variation of HBF group. (B) Coefficient of variation of LBF group. (C) Coefficient of variation of HIMF group. (D) Coefficient of variation of LIMF group.

Figure S3

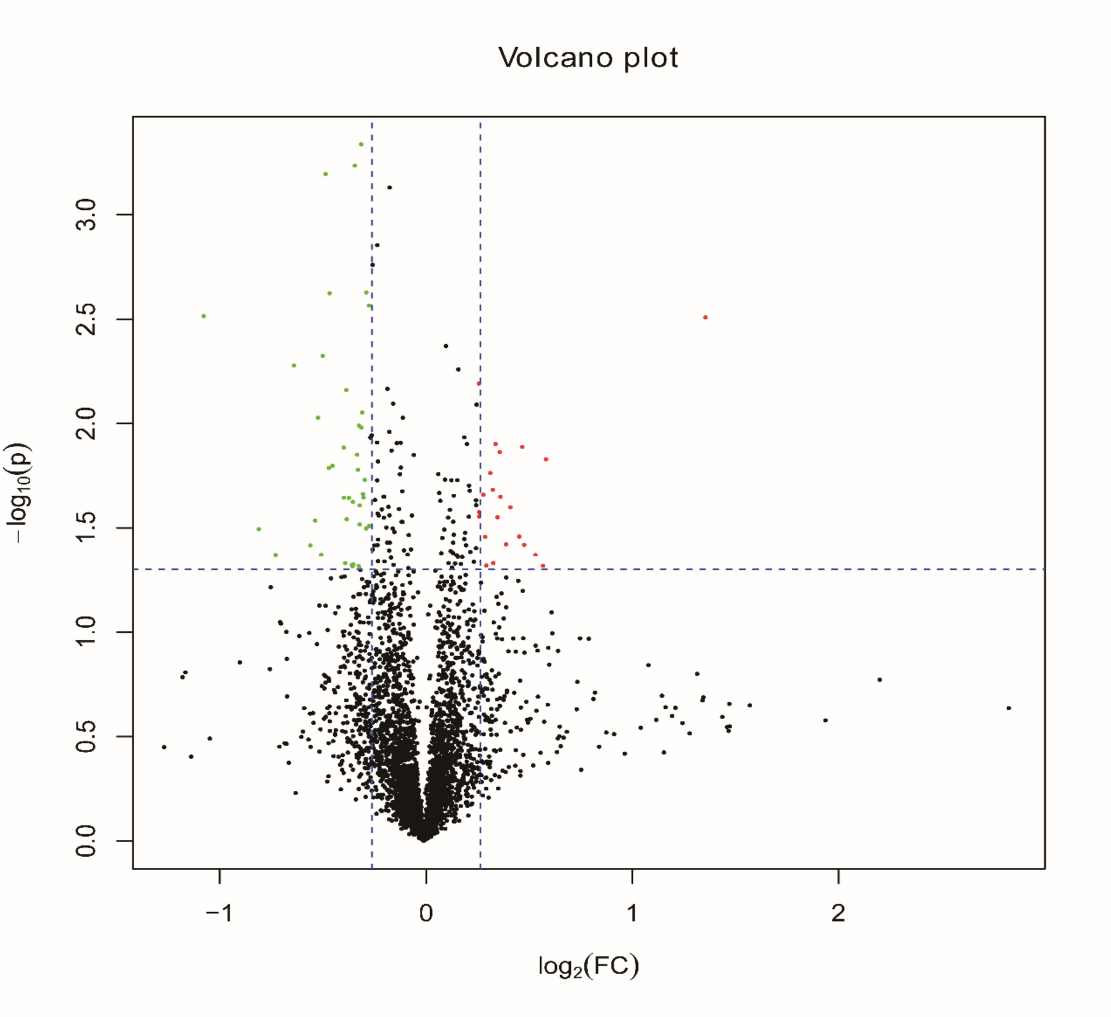

Figure S3. Volcano plot of DAPs from samples of subcutaneous fat. The abscissa indicates $\log_2$FC, the ordinate indicated -lg (*P*-value), red dots indicate differential expression of up-regulated proteins, green indicates differential expression of down-regulated proteins, and black indicates no differential expression.

Figure S4

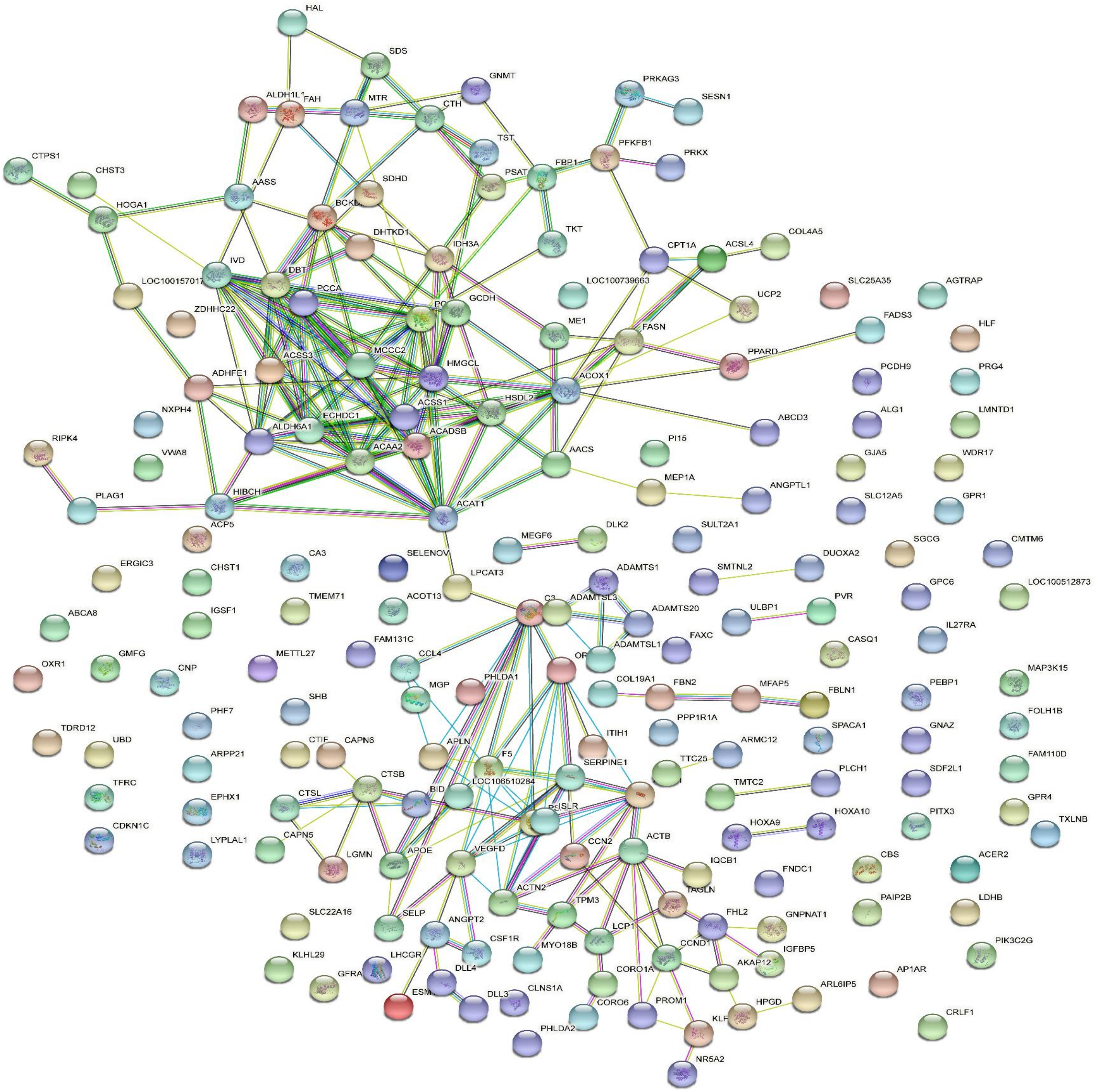

Figure S4. The interaction network diagram of both DEGs and DAPs in subcutaneous fat. The network nodes are proteins and the edges represent the predicted functional associations. Each colored lines represent different evidences for each interaction: red, gene fusions; green, gene neighborhood; blue, gene cooccurrence; purple, experimentally determined; yellow, textmining; light blue, from curated databases.

Figure S5

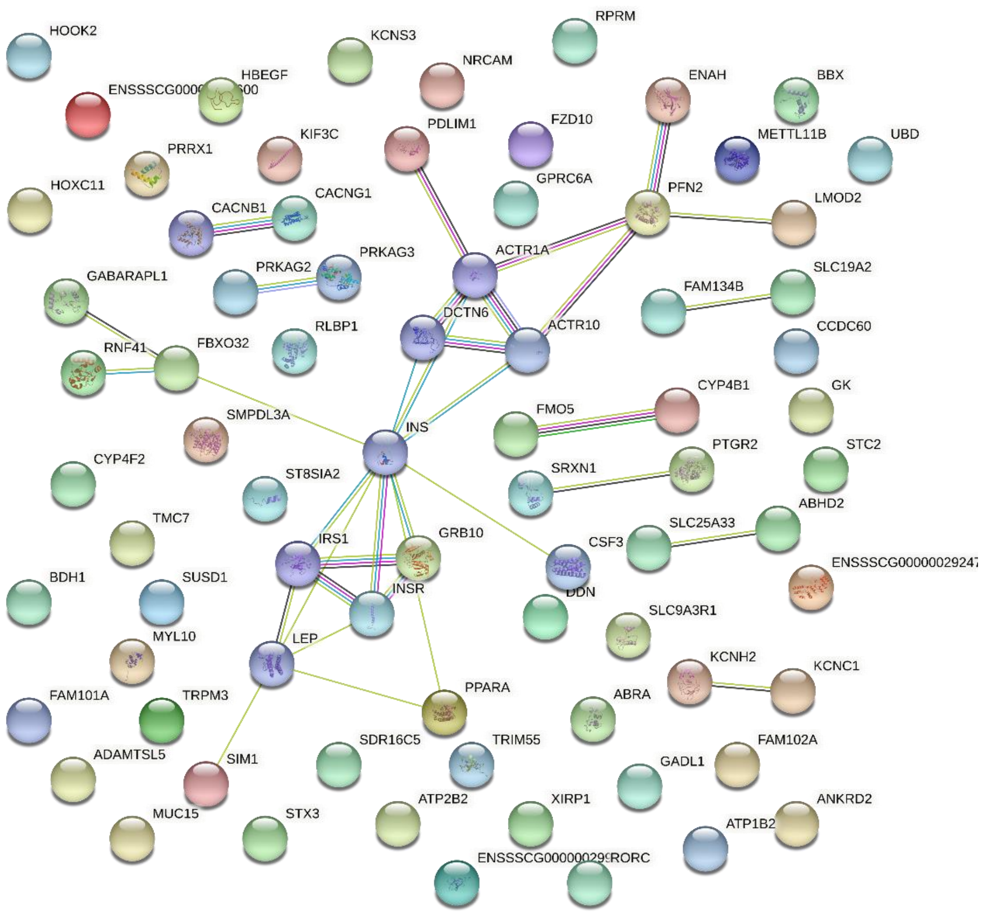

Figure S5. The interaction network diagram of both DEGs and DAPs in LD muscle. The network nodes are proteins and the edges represent the predicted functional associations. Each colored lines represent different evidences for each interaction: red, gene fusions; green, gene neighborhood; blue, gene cooccurrence; purple, experimentally determined; yellow, textmining; light blue, from curated databases.

Figure S6

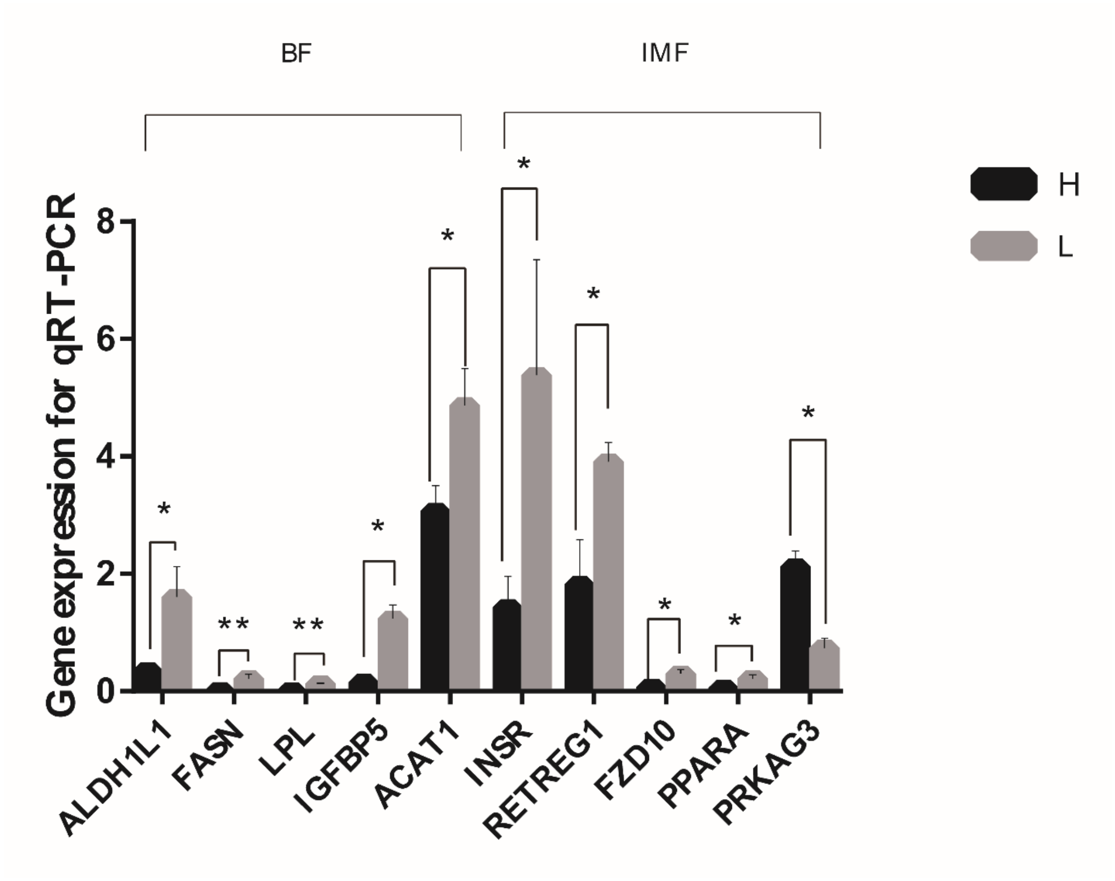

Figure S6. qPCR validation of 10 genes differentially expressed according to RNA-seq and TMT basic proteome .

*on the bars indicate significant differences ($P < 0.05$) and ** indicate extremely significant differences ($P < 0.01$) between HBF and LBF or HIMF and LIMF.
